# Integrins and p53 pathways in glioblastoma resistance to temozolomide

**DOI:** 10.3389/fonc.2012.00157

**Published:** 2012-10-31

**Authors:** Sophie Martin, Hana Janouskova, Monique Dontenwill

**Affiliations:** Laboratory of Biophotonics and Pharmacology, UMR 7213 CNRS, Faculté de Pharmacie, Université de StrasbourgIllkirch, France

**Keywords:** integrin, p53, temozolomide, glioblastoma, chemoresistance

## Abstract

Glioblastoma is the most common malignant primary brain tumor. Surgical resection, postoperative radiotherapy plus concomitant and adjuvant chemotherapy with temozolomide (TMZ) is the standard of care for newly diagnosed glioblastoma. In the past decade, efforts have been made to decipher genomic and core pathway alterations to identify clinically relevant glioblastoma subtypes. Based on these studies and more academic explorations, new potential therapeutic targets were found and several targeting agents were developed. Such molecules should hopefully overcome the resistance of glioblastoma to the current therapy. One of the hallmarks of glioblastoma subtypes was the enrichment of extracellular matrix/invasion-related genes. Integrins, which are cell adhesion molecules important in glioma cell migration/invasion and angiogenesis were one of those genes. Integrins seem to be pertinent therapeutic targets and antagonists recently reached the clinic. Although the p53 pathway appears often altered in glioblastoma, conflicting results can be found in the literature about the clinically relevant impact of the p53 status in the resistance to TMZ. Here, we will summarize the current knowledge on (1) integrin expression, (2) p53 status, and (3) relationship between integrins and p53 to discuss their potential impact on the resistance of glioblastoma to temozolomide.

## INTRODUCTION

Glioblastoma is characterized by rapidly dividing cells, high degree of vascularity, invasion into the normal brain tissue, and an intense resistance to death-inducing stimuli. Significant advances have been made in understanding the molecular genetics underlying the heterogeneity of glioblastoma and their resistance to therapies. However, standard therapy including surgical resection and radiotherapy with concomitant and adjuvant chemotherapy using temozolomide (TMZ) remains poorly efficient (Stupp et al., 2005). Prognostic and predictive markers are continuously proposed based on large scale genomic data. In the recent years, emphasis has been given to the predictive impact of O^6^-methylguanine-DNA methyltransferase (MGMT) expression/activity, a DNA repair enzyme that protects cells against alkylating drugs such as TMZ (Stupp et al., 2009). The promoter of the MGMT gene is methylated in 40–45% of glioblastoma and the enzyme is not expressed in the majority of these cases (Hegi et al., 2005; Silber et al., 2012). While the contribution of MGMT to TMZ resistance is highly documented, tumors in which MGMT is not the primary determinant of treatment outcome also exist (Carlson et al., 2009; Combs et al., 2011). Integrins have been proposed to play a role in the aggressiveness of gliomas and have been implicated in radio/chemoresistance in different types of tumors (Aoudjit and Vuori, 2012). The p53 protein has been largely studied in gliomas but its prognostic value has not been consistently established. In line with our recent data proposing an α_5_β_1_ integrin-p53 axis with potential implication in TMZ resistance (Janouskova et al., 2012), we will summarize here the current knowledge on integrins and p53 status in glioblastoma.

## INTEGRINS IN GLIOMA

### BIOLOGY OF INTEGRINS

Integrins are heterodimeric cell surface receptors that mediate cell adhesion to the extracellular matrix (ECM) and support cell–cell interactions in a multitude of physiological and pathological situations. They are at least 24 known αβ heterodimers formed by a combination of 18 α and 8 β subunits bound non-covalently. Natural ligands of integrins are component of the ECM such as vitronectin, collagen, or fibronectin. Each αβ integrin pair has a defined set of ECM protein (Hynes, 2002). The repertoire of integrins present at the membrane dictates therefore the extent to which a cell will behave on a specific matrix and respond to its environment. Once engaged with the ECM, integrins cluster and recruit various signaling and adaptor proteins to form focal adhesion complexes (Geiger et al., 2001). These complexes activate intracellular downstream signaling pathways including NF-κB, PI3K, Src, or Ras-MAP kinases (Hynes, 2002; Legate et al., 2009). Such pathways regulate functions involved in motility, cytoskeleton organization, adhesion, proliferation, survival, and gene transcription. Integrins link ECM to the actin cytoskeleton through FAK/ILK/SFK/Rho proteins pathway providing the traction necessary for cell motility (Geiger et al., 2001). Integrins regulate the localization and the activity of urokinase-type plasminogen activator (uPA)/uPA receptor (Ghosh et al., 2000; Wei et al., 2007; Bass and Ellis, 2009) and matrix metalloproteinases (MMPs; Lamar et al., 2008; Morozevich et al., 2009) therefore controlling ECM remodeling and the invasive process.

Beside their mechanical functions and despite the lack of intrinsic kinase activity, integrins are true signaling molecules. Integrins regulate proliferation by controlling the expression of cyclin D1 which permits cells to enter the S-phase of the cell cycle (Fournier et al., 2008). Integrins relay survival or apoptotic signals depending on the surrounding environment. Integrin ligation promote survival through various mechanisms including increased anti-apoptotic proteins (bcl-2, FLIP; Aoudjit and Vuori, 2001b; Matter and Ruoslahti, 2001; Uhm et al., 1999), activation of PI3K–Akt (Aoudjit and Vuori, 2001a) or NF-κB pathway (Scatena and Giachelli, 2002; Courter et al., 2005). Unligated integrins were reported to promote apoptosis through the so-called integrin-mediated death (IMD) a mechanism dependent, or not, on caspases activation in anchorage-dependent cells (Stupack et al., 2001; Jan et al., 2004). However, tumor cells are often IMD-resistant and unligated integrins rather promote anchorage-independent growth, survival, and metastasis than apoptosis (Desgrosellier et al., 2009). Additionally, cross-talks occur between integrins, cytokines, and growth factor receptors. Optimal growth factor stimulation relies on integrin-mediated adhesion to an appropriate ECM protein. α_v_β_3/5_ and α_5_β_1_ interact with growth factor receptors (VEGFR2, c-Met, FGFR1, PDGFR, EGFR, TIE-2, and IGF-1R) to promote full activation of each receptor and maximal signal transduction (increased MAPK and Akt activity) resulting in enhanced cell migration, proliferation, survival, and angiogenesis (Friedlander et al., 1995; Eliceiri, 2001; Alam et al., 2007; Soung et al., 2010). Integrins were also reported to bind directly growth factor (such as angiopoietins or VEGF) allowing the transduction of information in the absence of the receptor (Carlson et al., 2001; Hutchings et al., 2003). In short, integrins sense, interpret, and distribute information so that cancer cells adjust and respond to their microenvironment.

### INTEGRIN EXPRESSION AND FUNCTION IN GLIOMA

Clustering of transcriptomic data from high grade glioma predicted poor survival in subclasses of tumors overexpressing ECM components such as fibronectin which is the preferred ligand of α_5_β_1_ and α_v_β_3_ integrins (Geiger et al., 2001; Freije et al., 2004; Bredel et al., 2005; Colin et al., 2006; Tso et al., 2006). Functional analysis revealed gliomagenesis and glioblastoma networks composed of genes that play a role in integrin signaling including fibronectin, α_3_ and α_5_ integrins (Bredel et al., 2005). Gingras et al. (1995) investigated glioblastoma for the expression of cell adhesion molecules including integrins that might distinguish tumor from normal adjacent brain tissue. Results showed that glioblastoma expressed α_2_, α_3_, α_5_, α_6_β_1_, and α_v_β_3_ integrins at significantly higher level than normal brain tissue suggesting that these integrins might play a role in the development or the progression of glioma (Gingras et al., 1995). β_8_ and α_5_β_1_ integrins were commonly expressed in a perinecrotic or perivascular pattern in glioblastoma (Riemenschneider et al., 2005). Higher levels of α_5_ and β_3_ integrin mRNA were measured in glioblastoma as compared to normal brain or low grade astrocytoma (Kita et al., 2001). Average α_v_β_3_ integrin expression in glioblastoma seemed to exceed those in low grade glioma at the protein level although mRNA levels of both subunits were not discriminative between glioblastoma and low grade glioma (Schnell et al., 2008). In another study, α_v_β_5_ and α_5_β_1_ integrins were shown to be expressed at consistently higher levels than α_v_β_3_ integrins in human glioma cell explants (Mattern et al., 2005). We and others showed recently that α_5_β_1_ integrin expression in biopsies from patient with glioma correlated with poor prognosis and tumor aggressiveness (Cosset et al., 2012; Holmes et al., 2012; Janouskova et al., 2012).

In glioma, integrins were often studied because of their crucial role in tumor cell invasion (Gritsenko et al., 2012). Both β_1_ subunit-containing integrins (Paulus et al., 1996) and α_v_β_3_ integrins control glioma cell invasion (D’Abaco and Kaye, 2007). α_2_β_1,_ α_3_β_1_, and α_5_β_1_ integrins are overexpressed in multidrug-resistant glioma cells and are responsible for their increased adhesive and invasive capacities (Hikawa et al., 2000). The laminin-5 receptor α_3_β_1_ integrin is mainly expressed in areas of tumor cell invasion and support glioma cell migration and invasion (Tysnes et al., 1996; Mahesparan et al., 2003; Kawataki et al., 2007). α_v_β_3_ integrins promote migration and adhesion in various glioma cells (Gladson and Cheresh, 1991; Friedlander et al., 1996) and inhibition of α_v_β_3_ integrin with neutralizing antibody inhibited migration and invasion selectively in cell lines that contained a high level of integrin expression (Wild-Bode et al., 2001). Our laboratory extensively investigated α_5_β_1_ integrins in glioma. We showed that α_5_β_1_ integrins increased proliferation, clonogenic survival, adhesion, migration, and invasion of various glioma cell lines (Maglott et al., 2006; Bartik et al., 2008; Martin et al., 2009; Cosset et al., 2012). We also reported that the expression of α_5_β_1_ integrins in glioma is controlled by caveolin-1 (Martin et al., 2009; Cosset et al., 2012). Interestingly, it was shown recently that invasive recurrent glioblastoma, resistant to antiangiogenic therapy, overexpress α_5_β_1_ integrin and its ligand fibronectin (DeLay et al., 2012). α_v_β_3_ integrin/ILK/RhoB pathway (Monferran et al., 2008) and β_1_ integrin/AKT/p130Cas/paxillin (Cordes et al., 2006) controlled the radiosensitivity of glioma cells by regulating radiation-induced cell death. Recruitment of α_v_β_3/5_ integrin in glioblastoma cells is induced by hypoxia. It follows the activation of FAK/RhoB/GSK3β pathway leading to HIF-1α induction and the transcription of proangiogenic factors (Skuli et al., 2009). Finally, surface expression of α_6_β_1_ integrin in U87MG cells enhanced cell spreading and attachment on laminin-111, increased proliferation, decreased apoptosis due to serum starvation and increased migration and invasion of U87MG cells both *in vitro* and *in vivo* (Delamarre et al., 2009).

### INTEGRINS IN GLIOMA ANGIOGENESIS

Induction of angiogenesis is essential for a tumor to grow beyond 1–2 mm and glioblastoma exhibit prolific angiogenesis. Poorly expressed in resting endothelial cells, α_5_β_1_ and α_v_β_3/5_ integrins are highly upregulated on endothelium cells during tumor angiogenesis (Bussolati et al., 2003; Avraamides et al., 2008; Desgrosellier and Cheresh, 2010) and rapidly accessible in tumor blood vessels (Magnussen et al., 2005). They stimulate endothelial cell proliferation, promote migration, and lumen formation (Mettouchi and Meneguzzi, 2006). Although α_5_β_1_ integrin is undoubtedly recognized as a proangiogenic factor, controversial results for αvβ3 integrin questioned its role (Hodivala-Dilke, 2008; Robinson and Hodivala-Dilke, 2011). Overexpression of α_v_β_3_ integrin in glioma exerted growth-suppressive effects *in vivo* that are linked to vascular defects (Reynolds et al., 2002; Kanamori et al., 2004, 2006). However, antagonists to both α_5_β_1_ and α_v_β_3/5_ integrins are able to inhibit tumor angiogenesis (Brooks et al., 1995; Friedlander et al., 1995; Kim et al., 2000).

### INTEGRINS IN GLIOMA STEM CELLS

Brain tumors also contain highly tumorigenic and therapeutically resistant pluripotent stem cells referred as glioma stem or initiating cells. The glioma stem cell hypothesis incorporates a model in which only a small subset of cells, the glioma stem cells, can initiate tumor. This hypothesis was confirmed very recently *in vivo* (Lathia et al., 2011). Elevated levels of α_6_β_1_ integrins were found in glioma stem cells and seem to be a reliable new marker to enrich for glioma stem cells (Lathia et al., 2010).

Integrins are implicated at various levels of glioma development and progression. Blocking their functions may affect both tumoral cells and endothelial cells and these characteristics made them attractive therapeutic targets for glioblastoma (Chamberlain et al., 2012; Goodman and Picard, 2012). Emphasis on α_v_β_3_ integrins has been given recently as cilengitide, their prototypical small peptide antagonist, is currently evaluated in phase III clinical trials in glioblastoma (Tabatabai et al., 2010). Interestingly, the outcome in a phase II trial was particularly good in patients with a methylated MGMT gene promoter (Stupp et al., 2010). Emerging data showing the role of α_5_β_1_ integrin in glioblastoma give some hope for new therapeutic propositions in the near future.

## p53 PROTEIN IN GLIOMA

p53, the “guardian of the genome,” is certainly one of the most widely studied protein in human glioma. Activation of the tumor suppressor p53 by stress signals triggers different cellular programs such as cell cycle arrest, apoptosis, differentiation, DNA repair, autophagy, and senescence through complex network and signaling pathways (Levine and Oren, 2009; Vousden and Prives, 2009; Sullivan et al., 2012). Gaining a better understanding of how transcriptional and non-transcriptional functions of p53 integrate will be of great importance for the proposal of new therapeutic options (Dai and Gu, 2010; Speidel, 2010). Somatic p53 missense mutations are found in approximately 50% of all human cancers. Intensive research on p53 status as a classical molecular marker led to controversial results and non-significant clinical impact, particularly in the glioma field.

### p53 STATUS IN GLIOMA

As most mutations in p53 gene led to the accumulation of p53 in the nucleus, nuclear overexpression of p53 was usually considered as a marker of mutation. Several studies showed that the expression of p53 is correlated at 90% with its mutation (Figarella-Branger et al., 2011). Detection of p53 mutation by the yeast functional assay that measures quantitatively mutant p53 alleles and qualitatively the loss of p53 competence was also employed and compared to conventional techniques including DNA sequencing (Tada et al., 1997; Fulci et al., 2000). Overall results indicate that p53 mutations often occurred in low grade gliomas (WHO grade II astrocytoma; Bourne and Schiff, 2010) and thus is a frequent event in the pathological progression of secondary glioblastoma (WHO Grade IV; Gladson et al., 2010). Secondary glioblastoma arise from a preexisting grade II or III astrocytoma in contrast with primary glioblastoma that form *de novo*. Primary glioblastoma represent about 90% of glioblastoma. p53 gene mutations are present in about 30% of primary glioblastoma, and occur more frequently in secondary glioblastoma (65%; Ohgaki et al., 2004; Zheng et al., 2008). A recent integrated genomic analysis identified four relevant subclasses of glioblastoma (proneural, mesenchymal, neural, and classical glioblastoma). p53 mutation was observed in 54, 32, 21, and 0% of tumors from the proneural, mesenchymal, neural, and classical glioblastoma subtype, respectively (Verhaak et al., 2010). Interestingly, classical glioblastoma benefit from more aggressive therapy regimen than the others (Verhaak et al., 2010). In fact, the prognostic value of p53 status may be reconsidered according to these data.

### p53 STATUS AND GLIOBLASTOMA PROGNOSIS

No clear consensus has been reached about the prognostic value of p53 status despite numerous studies (**Table 1**). A clear picture remains difficult to draw due to the different techniques used to evaluate p53 (including immunostaining on tumor tissues, direct sequencing of p53 gene, and functional assays) and the complexity of patient cohort composition. Data illustrating an association of p53 with survival always point to a longer survival when p53 is mutated (Tada et al., 1998; Schiebe et al., 2000; Birner et al., 2002; Burton et al., 2002). However, the majority of studies do not validate p53 as an independent prognostic marker for glioblastoma (Kraus et al., 2001; Simmons et al., 2001; Shiraishi et al., 2002; Rich et al., 2005; Ruano et al., 2009; Weller et al., 2009; Levidou et al., 2010; Rossi et al., 2011). Overall it means that the prognostic impact of p53 aberrations is only marginal when considered in a global glioblastoma patient population. Reevaluation of this impact in clinically relevant glioblastoma subpopulations (see above) and association with specific molecular signatures will certainly be of interest in the future.

**Table 1:** Evaluation of p53 status in glioblastoma.

Evaluation of the p53 status	Number of patients	% of p53 mutant	p53: prognostic marker?	Reference
Sequencing/yeast functional assay	42	43	YES (longer survival for patients with p53mut tumors)	Tada et al. (1998)
Sequencing	75	32	YES (longer survival for patients with p53mut tumors)	Schiebe et al. (2000)
Sequencing/immunostaining	110	19	NO	Simmons et al. (2001)
Sequencing/immunostaining	93	22	NO	Kraus et al. (2001)
Sequencing/yeast functional assay	123	31	NO	Shiraishi et al. (2002)
Sequencing/immunostaining	41 long-term survivors	25	YES (longer survival for patients with p53 positive tumors)	Burton et al. (2002)
	48 short-term survivors	31		
Immunostaining	114	/	YES (longer survival for patients with p53 positive tumors)	Birner et al. (2002)
Sequencing	41	27	NO	Rich et al. (2005)
Sequencing/immunostaining	194	/	NO	Ruano et al. (2009)
Sequencing/immunostaining	291	15	NO	Weller et al. (2009)
Immunostaining	77 Meta analysis	/	NO	Levidou et al. (2010)
Immunostaining	106	/	NO	Rossi et al. (2011)

### p53 AND GLIOMA-INITIATING STEM CELLS

Recent studies begin to shed light onto the role of p53 in the regulation of neural stem cells (NSCs). NSCs are self-renewing cells in the central nervous system that can generate both neurons and glia. An elegant study showed that dual inactivation of p53 and PTEN in murine NSC promotes an undifferentiated state with high renewal potential and generates tumors with a high grade glioma phenotype (Zheng et al., 2008). Although the role of p53 in brain tumor stem cells has not been well established, data suggest that loss of differentiation and increase in neurosphere renewal may be linked to the disruption of the p53 pathway in glioma (Molchadsky et al., 2010; Mendrysa et al., 2011; Spike and Wahl, 2011). To achieve a permanent eradication of brain tumors, it is noteworthy that glioma-initiating stem cells have to be considered and in this way their p53 status and functions need to be further explored.

### p53 AND TMZ

Despite expressing mainly a wild-type p53 and thus being expected to be sensitive to DNA-damaging agents, primary glioblastoma resist standard therapies including chemotherapy with TMZ. This intriguing observation is in debate and the role of p53 status in response to TMZ has been largely addressed in preclinical studies. Conflicting results have been obtained (**Table 2**) and show either an improved capability of TMZ to inhibit cell viability when p53wt is functionally inhibited (Hirose et al., 2001; Xu et al., 2001, 2005a,b; Dinca et al., 2008; Blough et al., 2011) or a sensitization of cells to drugs when p53wt is functional (Hermisson et al., 2006; Roos et al., 2007). The former studies suggested that glioma cells with an intact p53 gene are selectively impaired in the proapoptotic functions of p53wt while retaining the potential to mediate relevant DNA repair and cell cycle arrest. Treatment with TMZ induced a persistent cell cycle arrest and an increase in p21 (a cell cycle regulator) in functional p53-expressing cells which showed morphological and biochemical features of senescent cells (Hirose et al., 2001; Martinkova et al., 2010). In cells impaired for p53 function or with a mutant p53, TMZ induced a transient cell cycle arrest and cell death via apoptosis or mitotic catastrophe (Hirose et al., 2001; Martinkova et al., 2010) as well as attenuation of DNA repair (Xu et al., 2005b). When TMZ-triggered apoptosis was reported for both p53wt and p53mutant cells, pathways involved differed with activation of the FAS apoptotic pathway or the mitochondrial apoptotic pathway, respectively (Roos et al., 2007). Thus adverse effects of p53wt activities are increasingly recognized and may participate in chemoresistance of diverse cancers including glioma (Kim et al., 2009; Martinez-Rivera and Siddik, 2012). In one recent report, the effect of p53 status on response to TMZ was explored in glioma-initiating stem cells. It was shown that tumor stem cells are resistant to TMZ when p53 is mutated and sensitive to TMZ when intact (Blough et al., 2011). These data add a new level of complexity in the relationship between p53 status and TMZ sensitivity in glioma.

**Table 2:** Role of p53 inTMZ outcome.

Material	p53 inhibition	Effect of p53 modulation onTMZ sensibility	Reference
Glioblastoma cell lines (U87MG, LNZ308)	By oncoprotein E6	Increased sensibility	Hirose et al. (2001)
Glioblastoma cell lines (SWB95, SWB77, SWB33,		p53-independent cell cycle arrest	Bocangel et al. (2002)
SWB40, SWB39, SWB61, D54) xenografts			
Glioblastoma cell lines (U87MG, LNZ308)	By pifithrin-α	Increased sensibility	Xu et al. (2005a)
Glioblastoma cell lines (D54, A172)	By oncoprotein E6	Increased sensibility	Xu et al. (2005b)
Glioblastoma cell lines (U87MG, U373MG, U251MG,	By siRNA	Decreased sensibility	Hermisson et al. (2006)
U138MG, LN18, LN428, LN319, LNT229, LN308,			
D247MG,T98G)			
Glioblastoma cell lines (U87MG, U138MG)	By pifithrin-α	Decreased sensibility	Roos et al. (2007)
Glioblastoma cell lines (U87MG) xenografts of biopsies	By pifithrin-α	Increased sensibility	Dinca et al. (2008)
Glioblastoma cell lines (U87MG, LNZ308, LN443, SF767,	By siRNA	In cell lines : increased sensibility	Blough et al. (2011)
U251N, U373)			
Cancer stem cells		In stem cells : decreased sensibility	

## INTEGRINS AND p53

Although p53 itself is functional in a great majority of primary glioblastoma, inactivation of the p53 signaling pathway occurred in the form of ARF deletions, amplifications of mdm2 or mdm4 leading to p53 signaling alterations in 87% of glioblastoma (Network, 2008). Additionally several oncogenes such as the glioma oncoprotein Bcl2L12 (Stegh et al., 2010) were reported to be overexpressed in p53wt tumors and to impair p53 signaling pathway. Few reports investigated the relationship between integrins and p53 signaling pathways. Both integrin-dependent activation (Lewis et al., 2002) and inhibition (Bao and Stromblad, 2004) of p53 signaling have been suggested in different tumoral settings except gliomas. For example, Stromblad and colleagues demonstrated that α_v_β_3_ integrin impacts negatively on p53wt activity in melanoma cells (Bao and Stromblad, 2004; Smith et al., 2012). We proposed recently that α_5_β_1_ integrin plays a similar role in high grade glioma (Janouskova et al., 2012). We demonstrated that overexpression of the α_5_ integrin subunit in p53wt U87MG cells impaired the activation of p53 and its transcriptional activity in response to TMZ. Under such conditions, cells became resistant to this alkylating agent. No such effects were found in p53 mutant glioma cell lines. Interestingly, higher levels of α_5_ integrin were found in p53wt tumor biopsies than in p53 mutant tumor biopsies suggesting a link between this specific integrin and p53 status *in vivo*. Our *in vitro* studies also demonstrated that SJ749 and K34c, two specific non-peptidic antagonists of α_5_β_1_ integrin, improved the therapeutic action of TMZ in a p53-dependent way (Martinkova et al., 2010). Molecular pathways involved in the integrin-dependent chemoresistance in p53wt tumors are currently unknown and deserve further studies. Proteins implicated in integrin signaling have been shown to shuttle between the membrane and the nucleus providing a potential mechanism for communication between integrins and p53. In particular FAK, the main kinase activated by integrins, is known to interfere with p53 activity in the nucleus (Lim et al., 2008; Golubovskaya and Cance, 2011). It will also be interesting to discriminate the potential effect of α_5_β_1_ integrin on transcriptional and non-transcriptional functions of p53. Our data are the first to demonstrate relationships between an integrin and the TMZ-dependent activation of p53 in glioma possibly explaining the resistance to TMZ of a subgroup of patients. It is tempting to suggest that inhibition of α_5_β_1_ integrin by specific antagonists might be an adjuvant treatment to standard therapies in patients expressing high level of α_5_β_1_ integrin and p53wt.

## CONCLUSION

Despite few and sometimes conflicting data available both on integrin expression and p53 status as prognostic and/or predictive markers for high grade glioma, a reevaluation of their roles is warranted. Due to the growing knowledge on glioblastoma heterogeneity and subclassification, it becomes reasonable to address these questions more accurately in well defined subpopulations of patients. Key issues need still to be addressed before proposing α_5_β_1_ integrin expression level and p53 status as relevant biomarkers to stratify group of patients which may be more responsive to TMZ.

## Conflict of Interest Statement

The authors declare that the research was conducted in the absence of any commercial or financial relationships that could be construed as a potential conflict of interest.
